# Leonurine ameliorates experimental type 2 diabetes through gut microbiota remodeling, enhanced butyrate production, and MPC2 activation to restore GLP-1 secretion

**DOI:** 10.3389/fphar.2026.1747267

**Published:** 2026-02-02

**Authors:** Yaoyuan Zhang, Wanyi Chen, Xinyuan Yu, Jianhua Feng, Abdul Sammad, Zhenbo Wang, Kai Yin

**Affiliations:** 1 Department of General Practice, The Fifth Affiliated Hospital of Southern Medical University, Guangzhou, Guangdong, China; 2 Guangxi Key Laboratory of Diabetic Systems Medicine, Guilin Medical University, Guilin, Guangxi, China; 3 State Key Laboratory of Bioactive Molecules and Druggability Assessment, Guangdong Province Key Laboratory of Pharmacodynamic Constituents of TCM and New Drugs Research, Institute of Traditional Chinese Medicine & Natural Products, College of Pharmacy, Jinan University, Guangzhou, China

**Keywords:** butyrate, diabetes, GLP-1, gut microbiota, leonurine, MPC2

## Abstract

The core pathophysiological mechanism of type 2 diabetes mellitus (T2DM) is closely associated with gut microbiota dysbiosis and its consequential impairment of enteroendocrine glucagon-like peptide-1 (GLP-1) secretion. T2DM mouse model was established using high-fat diet (HFD) feeding combined with streptozotocin (STZ) administration. Diabetic mice received 30 or 60 mg/kg of leonurine (LEO) via daily gavage for 12 weeks. Gut microbiota composition was profiled by metagenomic sequencing, fecal short chain fatty acids (SCFAs) concentrations were quantified via enzyme-linked immunosorbent assay (ELISA), and GLP-1 expression was assessed using oral glucose tolerance tests (OGTT), ELISA, and immunofluorescence. *In vitro*, high-glucose (25 mM)-challenged GLUTag enteroendocrine cells were employed to delineate the butyrate–mitochondrial pyruvate carrier 2 (MPC2) regulatory network using qPCR and Western blotting. LEO intervention significantly ameliorated glucose intolerance in diabetic mice and elevated GLP-1 levels in serum and colonic tissues. Metagenomic analysis revealed that LEO (60 mg/kg) remodeled gut microbiota structure, markedly enhancing α-diversity and specifically enriching butyrate-producing *Alistipes*. Mechanistically, butyrate activated MPC2 expression, effectively restoring cristae architecture defects observed by transmission electron microscopy, thereby promoting GLP-1 secretion. Crucially, MPC2 knockdown abrogated the secretagogue effect of butyrate on GLP-1 in GLUTag cells. LEO alleviates T2DM by remodeling the gut microbiota ecosystem, enhancing butyrate biosynthesis, and activating an MPC2-dependent mitochondrial energy metabolism pathway to reverse GLP-1 secretory dysfunction in intestinal L cells. This study establishes MPC2-mediated mitochondrial functional repair as a core mechanism through which microbial metabolites regulate enteroendocrine hormone secretion, identifying a novel therapeutic target within the “gut–islet axis” for diabetes intervention. Future studies should identify its active constituents, elucidate downstream effectors, and validate this mechanism in germ-free models.

## 1 Introduction

Type 2 diabetes mellitus (T2DM), characterized by insulin resistance and β-cell dysfunction, has emerged as a global health crisis with escalating prevalence (Mezza et al., 2019; Lu et al., 2024; Morieri et al., 2025). Current therapies, including insulin sensitizers and glucagon-like peptide-1 (GLP-1) receptor agonists (GLP-1RAs), face limitations such as hypoglycemia risk, weight gain, high costs, and poor patient adherence due to injectable administration (Sharma et al., 2018; Mezza et al., 2019; Chaiyakunapruk et al., 2025; Xie et al., 2025). These challenges underscore the urgent need for safe, cost-effective, and multitarget interventions. Recent advances highlight the gut microbiota and its metabolites as pivotal regulators of metabolic homeostasis, offering novel therapeutic avenues (Kriaa et al., 2019; Yang and Cong, 2021; Ji et al., 2025).

Dysbiosis of the gut microbiota is strongly associated with T2DM pathogenesis. Patients exhibit reduced microbial diversity, enrichment of opportunistic pathogens (e.g., Enterobacteriaceae), and depletion of short-chain fatty acid (SCFA)-producing taxa (e.g., *Bifidobacterium*, *Lactobacillus* (Cunningham et al., 2021; Murugesan et al., 2025; Zhang et al., 2025). SCFAs, including acetate, propionate, and butyrate, are fermentation products of dietary fibers that exert pleiotropic metabolic effects (Wang et al., 2024). Beyond serving as energy substrates, SCFAs activate G protein-coupled receptors (GPR41/43) on intestinal L cells, stimulating GLP-1 secretion to enhance insulin sensitivity and suppress hepatic gluconeogenesis (Kim et al., 2013; Lee et al., 2024). Additionally, SCFAs reinforce intestinal barrier integrity, mitigating endotoxemia-induced systemic inflammation—a key driver of insulin resistance (Kim et al., 2013; Liu et al., 2021; Zeng et al., 2024). These findings position microbiota remodeling to boost SCFA production as a promising strategy for T2DM.

GLP-1, a critical incretin hormone secreted by L cells, regulates glucose homeostasis via glucose-dependent insulin secretion and glucagon suppression. However, postprandial GLP-1 secretion is impaired in T2DM, exacerbating glycemic dysregulation. While synthetic GLP-1RAs (e.g., liraglutide) demonstrate efficacy, their high cost and invasive delivery limit accessibility (Lim and Brubaker, 2006; Lee et al., 2024; Chen et al., 2025). Thus, identifying natural compounds capable of enhancing endogenous GLP-1 release holds significant translational potential (Yaribeygi et al., 2021).

Leonurine (LEO, SCM-198), a major bioactive alkaloid derived from *Leonurus japonicus*, has garnered attention for its cardiometabolic benefits. *Leonurus japonicus* Houtt. is a traditional medicinal herb, widely used in Asia (Miao et al., 2019). The modern pharmacological profile of its principal alkaloid, LEO—encompasses anti-inflammatory, antioxidant, and microcirculation-improving activities (Shang et al., 2014; Miao et al., 2019). Early studies revealed its antioxidant and anti-inflammatory properties, contributing to neuroprotection and atherosclerosis mitigation. Recent evidence further demonstrates its capacity to modulate gut microbiota composition, rectifying homocysteine-methionine metabolic imbalance and attenuating cardiovascular risks (Liu et al., 2013; Jiang et al., 2017; Huang et al., 2021; Wang et al., 2025). Notably, LEO activates the METTL3/AKT1S1 axis to induce autophagy, ameliorating macrophage lipid accumulation and suppressing atherosclerotic plaque formation (Yu et al., 2024). These multi target effects of LEO suggests its potential in addressing metabolic disorders, such as T2DM.

The limitations of current therapies such as GLP-1 receptor agonists, and the role of microbial dysbiosis with its associated depletion of protective SCFAs. Therefore, this study aims to investigate the mechanisms of LEO intervention on the enrichment of SCFA-producing genera and suppression of pro-inflammatory microbiota. Furthermore, we aim to characterize the LEO’s effect on butyrate, a prominent SCFA, and a subsequent cascade of the stimulation of GLP-1 secretion from L cells, and its outcome on hyperglycemia and insulin resistance in T2DM model. Investigation to achieve the aims of this study involves employing well-established diabetic mouse model, metagenomics sequencing, SCFAs analysis, high glucose-induced GLUTag enteroendocrine cells, and loss-of-function studies. Henceforth, this study would evaluate LEO’s potential as a natural, orally administrable alternative to conventional therapies, a shift aimed at circumventing the inherent limitations of standard pharmaceutical approaches.

## 2 Materials and methods

### 2.1 Drugs and reagents

The reagents employed throughout this study included leonurine (20220301, Xinxiang Olan Biotechnology Co., Ltd), subsequently complemented by Ferrostatin-1 (Fer-1; HY-100579, MedChemExpress) and fetal bovine serum (FBS; M1043150S, BBI Solutions). Streptozotocin (STZ; S0132, Sigma-Aldrich) was systematically dissolved in citrate buffer (pH 4.5), while high-fat diet (HFD; 60% kcal fat, MD12032, Jiangsu MediSyn Biotechnology) served for metabolic induction. Concurrently, short-chain fatty acid standards (Sigma-Aldrich), GLP-1 ELISA kit (ab226922, Abcam), Oil Red O Staining Kit (G1262, Solarbio), RIPA Lysis Buffer (P0013B, Beyotime), TRIzol Reagent (15596026, Invitrogen), and anti-GLP-1 antibody (ab23468, Abcam) were utilized according to manufacturer specifications.

### 2.2 Mice and treatment

Eight-week-old male C57BL/6J mice sourced from Shanghai Slack Experimental Animals Co., Ltd (License number SCXK 2022-0004) were randomly allocated into four cohorts (n = 5/group). All mice were housed in a specific pathogen-free animal facility. The animals were fed normal chow diet or high fat diet (HFD) (Trophic Animal Feed High-Tech Co. Ltd., Nantong, China) and water *ad libitum* under a strict 12 h light/dark cycle. Control mice received standard chow with saline gavage. Experimentally, the type 2 diabetes (T2D) group underwent HFD feeding for 4 weeks followed by intraperitoneal STZ injections (50 mg/kg for 5 consecutive days) according to the protocol described in previous studies (Malla et al., 2015). Fasting blood glucose monitoring included measurements at 7 and 14 days after the last STZ injection. Diabetic status was confirmed by sustained fasting blood glucose >11.1 mmol/L under continued HFD. Serum fasting insulin levels were measured were estimated using an enzyme-linked immunosorbent assay (ELISA) kit (PI602, Beyotime Biotechnology). Furthermore, the homeostasis model assessment of insulin resistance (HOMA-IR) index was also calculated (Bowe et al., 2014). Subsequently, leonurine-treated groups (LEO) received daily oral gavage of 60 mg/kg leonurine post-confirmation. Throughout the 12-week protocol, body weight and glycemia were monitored weekly. Terminally, fecal samples were collected for microbiome/SCFA analysis alongside ileal tissue harvesting for histology.

### 2.3 Fecal microbiome analysis

Fresh fecal pellets were aseptically collected, immediately flash-frozen in liquid nitrogen, and preserved at −80 °C. Genomic DNA was subsequently extracted using the QIAamp Fast DNA Stool Mini Kit (51,604, Qiagen). Following library preparation, paired-end sequencing was performed on the Illumina NovaSeq platform targeting the 16S rRNA V3-V4 region. Bioinformatics analysis commenced with QIIME2-based OTU clustering, progressively advancing to α-diversity (Shannon index) and β-diversity assessments, ultimately employing Linear Discriminant Analysis (LDA) Effect Size (LEfSe) for differential taxon identification.

### 2.4 Fecal short-chain fatty acid quantification

SCFA concentrations were quantified using a commercial assay kit (K619-100, BioVision). Specifically, 50 mg feces were homogenized in 1 mL PBS containing 1% formic acid, followed by ice-bath sonication and centrifugation (12,000 × g, 15 min, 4 °C). The resulting supernatant was filtered (0.22 μm) and derivatized with DNPH under light-protected conditions for 30 min. Absorbance at 450 nm was measured spectrophotometrically (SpectraMax M5), with concentrations (μmol/g wet weight) calculated against standard curves (0–500 μM). Methodologically, blank controls and spiked recovery samples (85%–115% recovery) ensured analytical rigor.

### 2.5 Cell culture and treatment

GLUTag intestinal endocrine L-cells (Drucker et al., 1994) were routinely maintained in high-glucose DMEM supplemented with 10% FBS at 37 °C/5% CO_2_. For experiments, cells were treated as follows: Control (5.5 mM glucose), High Glucose (HG; 25 mM glucose, 24 h), HG + 40 μM leonurine (HG + LEO, 24 h), or HG + 2 mM sodium butyrate (HG + SCFA, 24 h).

### 2.6 Western blot analysis

The total proteins of colon tissues and cells were extracted by RIPA (#P0013C, Beyotime, China) with phosphatase inhibitor and PMSF (#P0012S, Beyotime, China). Subsequently, 30 μg protein per sample was electrophoresed by SDS-PAGE and transferred to PVDF membranes. Membranes were sequentially incubated with primary antibodies (anti-GLP-1 [Santa Cruz, sc-80603, 1:1,000], anti-MPC2 [Proteintech, 20049-1-AP, 1:2000], anti-GAPDH [Cell Signaling, D4C6R-97166, 1:5,000]) overnight at 4 °C, followed by HRP-conjugated secondary antibodies (Santa Cruz, sc-2354, 1:5,000) for 1 h at RT. Ultimately, protein bands were visualized by ECL-chemiluminescent kit (WBKLS0100, Millipore, United States) and quantified densitometrically using ImageJ.

### 2.7 RNA extraction and qRT-PCR

Quantitative real-time polymerase chain reaction (qRT-PCR) was carried out according to our previous published protocol (Zhang et al., 2025). In brief, total RNA was isolated with TRIzol reagent, with purity verified spectrophotometrically (A260/A280 > 1.8). Complementary DNA was synthesized using the PrimeScript RT Kit (RR037A, Takara). Subsequently, qRT-PCR was performed with SYBR Green Master Mix (RR820A, Takara), wherein *GLP-1* and *MPC2* expression levels were normalized to *GAPDH* and calculated via the 2^−ΔΔCT^ method. Primers used are given as: GAPDH (Mouse) forward sequence: AGG​TCG​GTG​TGA​ACG​GAT​TTG, and reverse sequence: GGG​GTC​GTT​GAT​GGC​AAC​A; GLP-1R (Mouse) forward sequence: GGG​TCT​CTG​GCT​ACA​TAA​GGA​CAA​C, and reverse sequence: AAG​GAT​GGC​TGA​AGC​GAT​GAC; MPC2 (Mouse) forward sequence: CCG​CCG​CGA​TGG​CAG​CTG, and reverse sequence: GCT​AGT​CCA​GCA​CAC​ACC​AAT​CC; GAPDH (Human) forward sequence: GCA​CAG​TCA​AGG​CCG​AGA​AT, and reverse sequence: GCC​TTC​TCC​ATG​GTG​GTG​AA; MPC2 (Human) forward sequence: TAC​CAC​CGG​CTC​CTC​GAT​AAA, and reverse sequence: TAT​CAG​CCA​ATC​CAG​CAC​ACA.

### 2.8 Histological staining

Ileal tissues underwent 4% paraformaldehyde fixation followed by paraffin embedding and sectioning (5 μm) for H&E staining to evaluate villus architecture. Alternatively, frozen sections (8 μm) were stained with Oil Red O solution for lipid droplet visualization, subsequently counterstained with hematoxylin. Quantitatively, lipid deposition area was determined using ImageJ.

### 2.9 Immunofluorescence staining

Colon cryosections were fixed and permeabilized with 0.1% Triton X-100. After blocking, sections were incubated overnight at 4 °C with anti-GLP-1 (1:200), followed by incubation with Alexa Fluor 594-conjugated secondary antibody (1:500) under light protection. Nuclei were counterstained with DAPI prior to imaging on a Leica TCS SP8 confocal microscope. Finally, fluorescence intensity was quantified using ImageJ.

### 2.10 Statistical analysis

Results are presented as the mean ± SEM. Statistical analysis was performed using GraphPad prism 9.0 software (9.0, GraphPad Software, San Diego, CA, United States). For data that followed a normal distribution (Shapiro-Wilk test), groups were compared using an unpaired, two-tailed Student’s t-test. For data that violated the normality assumption, the non-parametric Mann-Whitney U test was used. For comparison of three or more groups, one-way analysis of variance (ANOVA) followed by Tukey’s multiple comparisons test. A p-value <0.05 was selected as the threshold for statistical significance. All experiments were performed at least three times.

## 3 Results

### 3.1 Effect of LEO on OGTT and GLP-1 of diabetic mice

**FIGURE 1:**
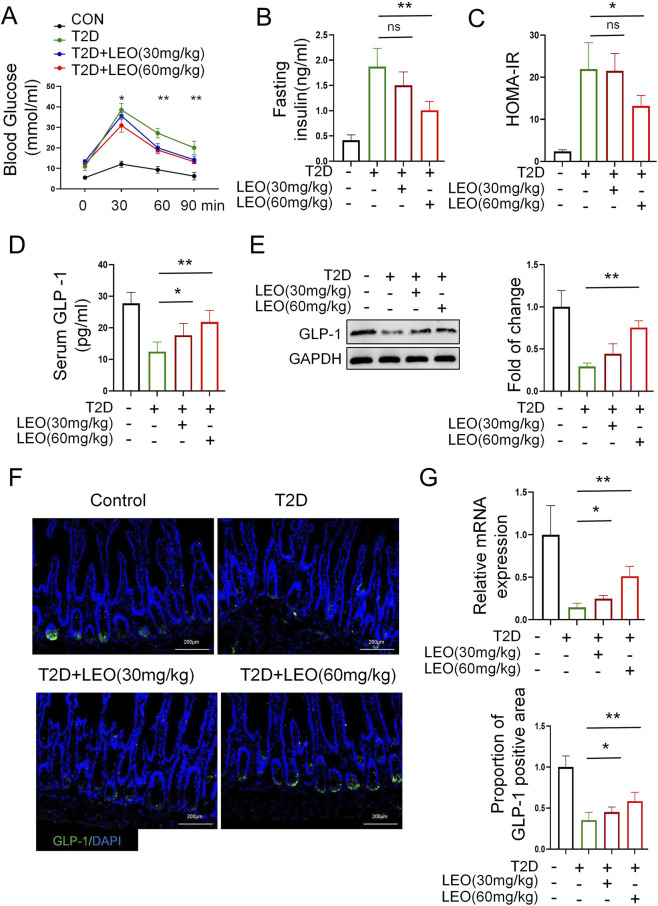
Leonurine (LEO) intervention improves glucose tolerance and enhances enteroendocrine glucagon-like peptide-1 (GLP-1) secretion in diabetic mice (T2D). **(A)** Glucose tolerance measured by oral glucose tolerance test (OGTT) at week 20 in mice (n = 5). **(B)** Fasting serum insulin content and **(C)** homeostasis model assessment-insulin resistance (HOMA-IR) in mice (n = 5) **(D)** GLP-1 levels were measured using an ELISA (Enzyme-Linked Immunosorbent Assay) kit in serum (n = 5) **(E)** Representative Western blot of GLP-1 from colon samples and quantification. **(F)** quantitative real-time polymerase chain reaction (qRT-PCR) analysis of the expression of the Gcg gene in colon samples. **(G)** Immunofluorescence staining of GLP-1 for colon samples and analysis of the proportion of GLP-1 positive area using ImageJ. The data represents at least three independent biological replicates (n ≥ 3), each containing three technical replicates. The data is presented as mean ± SD; Use independent sample t-test (or one-way ANOVA) for data that conforms to normal distribution; Non normally distributed data are analyzed using Mann Whitney U test (or Kruskal Wallis test) p < 0.05, **: p < 0.01, ***: p < 0.001.

We established an animal model of T2D by IP injection of STZ (50 mg/kg) and fed a HFD. Mice weight in the control group decreased from 30 ± 0.5 g to 26 ± 0.3 g, while in LEO (60 mg/kg) group it dropped to 24 ± 0.3 g. Fasting blood glucose levels were higher in both STZ and STZ + LEO mice than in the control group (no STZ injection). Twelve weeks after the intervention, oral glucose tolerance test (OGTT) was performed as shown in Figure 1A. The blood glucose of each group peaked 30 min after intragastric administration, and the blood glucose level of T2D was significantly higher than that of the other groups. Compared with the T2D group, blood glucose in the LEO (30 mg/kg) group mice and LEO (60 mg/kg) group mice decreased to different degrees after 1 h later, among which the blood glucose in the LEO (60 mg/kg) group decreased most significantly showing a dose-dependent decrease. These results are intriguing, as our previous studies did not demonstrate a significant reduction in blood sugar levels in OGTT involving intra-abdominal injection of glucose. Decrease in serum insulin level shows improved insulin sensitivity under metabolic stress (Chaour et al., 1997); where LEO (60 mg/kg) significantly decreased insulin levels (Figure 1B). Furthermore, an important systemic insulin resistance index, the HOMA-IR, was also significantly decreased in LEO (60 mg/kg) treated mice group (Figure 1C). The content of serum GLP-1 of the mice in the T2D group was significantly lower than that of the mice in the Control group (p < 0.01) (Figure 1D), while the intervention of LEO (30 mg/kg) and (60 mg/kg) could increase the content of serum GLP-1 of diabetic mice and showed dose-dependence. In addition, the expression of GLP-1 in the colon of diabetic mice was increased dramatically by the treatment of LEO at the doses of 30 mg/kg and 60 mg/kg as depicted in Figure 1E. GLP-1 immunofluorescence staining was performed to detect the expression of which in colon. As showed in Figure 1F, GLP-1 was decreased significantly in T2D group. While LEO treatment promoted GLP-1 protein expression to markedly higher levels than observed in the STZ-induced T2D group (Figure 1G). Collectively, these results indicate that LEO exerts a protective effect by increasing GLP-1 in mice with T2D.

### 3.2 Effect of LEO on gut microbiota dysbiosis of diabetic mice

**FIGURE 2:**
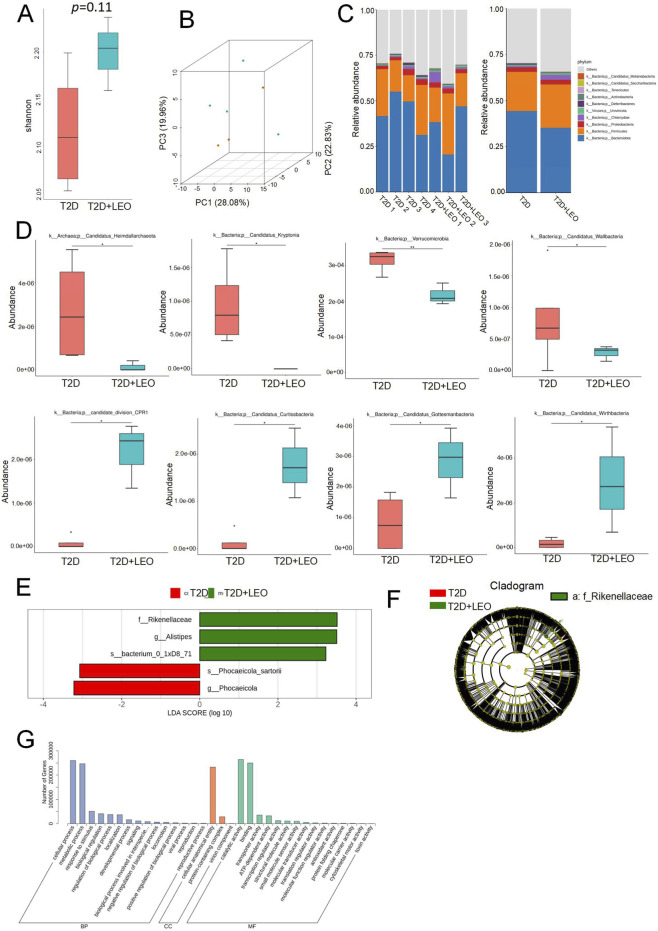
Leonurine (LEO) restores gut microbial ecology in diabetic mice (T2D) group (T2D + LEO): Enrichment of butyrogenic Alistipes and α-diversity recovery. **(A)** Shannon diversity index of fecal microbiota. **(B)** Principal Coordinate Analysis (PCoA) of microbiota composition. **(C)** Relative abundance of dominant microbial taxa (phylum/family level). **(D)** Differential abundance of specific bacterial taxa. **(E)** Using linear discriminant analysis (LEfSe, LDA>3.0), differential OTUs responsible for the discrimination between two groups were identified. **(F)** Partial Least Squares Discriminant Analysis (PLS-DA) calculated using Unweighted_unifrac at the OTU-level significantly separated. **(G)** Analysis and enrichment of gene ontology (GO) signaling pathways.

Next, the alterations in the gut microbiota of diabetic mice induced by LEO were investigated to further examine the effect of LEO on the composition of the gut microbiota in diabetic mice. Before the metagenomic sequencing, we selected the LEO (60 mg/kg) treatment group to perform the following experiments because the LEO (60 mg/kg) group had a better GLP-1 improvement effect than the LEO (30 mg/kg) group. The α-diversity changes in the intestinal microbes were assessed by using the Shannon Figure 2A. Compared with T2DM mice, LEO treated group showed a significant increase in the Simpson index, indicating LEO could elevate the copiousness and diversity of the intestinal gut microbiome. We used the principal component analysis (PCA) of the microbiota composition of the mice based on the weight UniFrac algorithm to compare the degree of variation across comparison of the overall microbiota structure Figure 2B. The distance between T2D and LEO treated group in the PCA plot stand for the level of similarity between the two groups. As shown in Figure 2C, each group of samples could be separated indicating that is different. In addition, a Histogram of relative abundance at the bacterial phylum level analysis displayed the entirety of the OTUs and the OTUs that overlapped within each group (Figure 2E). Our results identified at the phylum level, the relative percentage of Firmicutes was elevated by LEO, whereas the proportion of Bacteroidota and Deferribacteres was reduced compared with T2D group. In addition, by using metastats analysis, the LEO treatment group showed an increase in p_candidate_division_CPR1, Curtissbacteria, Gottesmanbacteria and Wirthbacteria compared to T2D group. In contrast, LEO treatment decreased the levels of Heimdallarchaeota, Kryptonia, Verrucomicrobia and Wallbacteria. (Figure 2D). The constitution and alterations of the intestinal microbes at the genus and species levels were further examined. The constitution and alterations of the intestinal microbes at the genus and species levels were further examined. Striking differential abundance patterns were observed between the LEO-treated cohort and the T2D control group (Figure 2F). The LEO intervention group exhibited a marked elevation in the relative abundance of *Alistipes* (genus-level) and the uncultured candidate species bacterium_0_1xD8_71, whereas a concurrent reduction was detected in *Phocaeicola sartorii* (species-level) and *Phocaeicola* (genus-level) populations. Subsequent Gene Ontology (GO) enrichment analysis delineated marked perturbations in key biological processes, including cellular processes (e.g., membrane integrity maintenance), metabolic reprogramming (particularly carbohydrate and lipid metabolism), cellular anatomical entity organization (notably gut epithelial junction complexes), catalytic activity modulation (encompassing oxidoreductases and hydrolases), and molecular binding dynamics (e.g., receptor-ligand interactions) (Figure 2G). In general, these findings provide additional evidence of the influence of LEO on modulating the community and constitution of the gut microbiota in STZ-induced T2D mice.

### 3.3 LEO elevates fecal SCFAs levels in diabetic mice: stimulation of GLP-1 secretion by altered SCFAs in GLUTag cells

**FIGURE 3:**
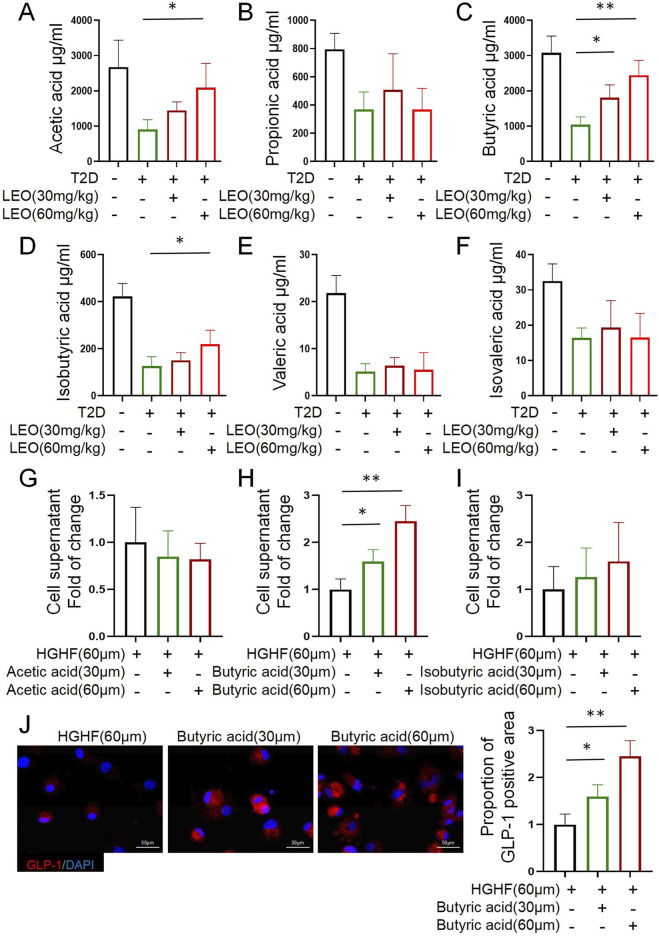
Fecal butyrate accumulation mediates glucagon-like peptide-1 (GLP-1) potentiation: Dose-responsive stimulation in high-glucose (HG)-stressed GLUTag cells. **(A–F)** Short-chain fatty acid (SCFA) concentrations (μg/mL) in colon contents: Acetic, Propionic, Butyric, Isobutyric, Valeric, and Isovaleric acids. **(G–I)** Fold change in GLP-1 secretion from cell supernatants treated with HG (60 μM) or SCFAs (30/60 μM). **(J)** Immunofluorescence of GLP-1 (green) and DAPI (blue) in cells exposed to butyric acid. Scale bars: 50 μm. The data represents at least three independent biological replicates (n ≥ 3), each containing three technical replicates. The data is presented as mean ± SD; Use independent sample t-test (or one-way ANOVA) for data that conforms to normal distribution; Non normally distributed data are analyzed using Mann Whitney U test (or Kruskal Wallis test) p < 0.05, **: p < 0.01, ***: p < 0.001.

We investigated the influence of LEO on the gut microbiota and its subsequent effects on the regulation of host metabolism. Oral administration of LEO significantly altered the fecal SCFA composition in diabetic mice in a dose-dependent manner. Notably, high-dose LEO (60 mg/kg) markedly elevated acetic acid and isobutyric acid levels, whereas butyric acid exhibited a robust increase even at the lower dose (30 mg/kg), with further enhancement at 60 mg/kg (Figures 3A–F). In contrast, propionic acid, valeric acid, and isovaleric acid remained unchanged across all treatment groups. It shall be noted that targeted metabolomics based measurements of SCFAs are more robust then the ELISA based measurements of ours. Anyway, this selective modulation of specific SCFAs—particularly the dose-responsive elevation of butyrate—suggests a potential mechanism by which LEO may ameliorate diabetic metabolic dysregulation, given the established role of butyrate in promoting gut integrity and insulin sensitivity.

To functionally link the altered SCFAs to GLP-1 secretion, we employed GLUTag cells under high-glucose (HG) conditions to mimic diabetic enteroendocrine dysfunction. Strikingly, among the three LEO-upregulated SCFAs (acetic acid, isobutyric acid, butyric acid), only butyrate stimulated GLP-1 secretion in a concentration-dependent manner (30 μM and 60 μM; *p* <0.01 vs. HG control; Figures 3G–I). This effect was further corroborated by immunofluorescence staining, which revealed intensified GLP-1 signal in butyrate-treated GLUTag cells (Figure 3J), indicating enhanced GLP-1 production and/or storage. Collectively, these findings establish a mechanistic pathway whereby LEO restores dysregulated SCFA production in diabetic mice, highlighting dose-responsive butyrate accumulation as the dominant effector mediating enhanced GLP-1 secretion in functionally compromised enteroendocrine cells.

### 3.4 MPC2 mediates butyrate-driven mitochondrial repair in LEO-treated diabetic mice

**FIGURE 4:**
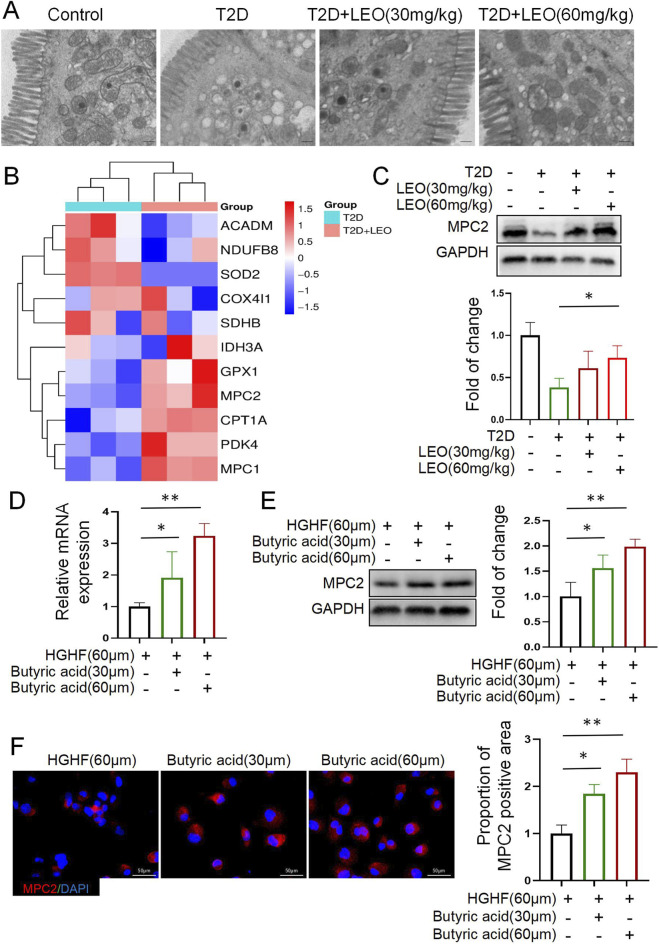
Mitochondrial structural-functional repair by butyrate-activated mitochondrial pyruvate carrier 2 (MPC2) in Leonurine (LEO)-treated colon. **(A)** Results of transmission electron microscopy (TEM) scanning showing mitochondrial morphologies (n = 8 per group) with scale bars, 500 nm. **(B)** Heatmap mainly showing expression levels of upregulated and downregulated ferroptosis-related genes. **(C)** Western blot of MPC2 protein in pancreatic tissues. **(D)** Fold change in MPC2 mRNA expression in pancreatic tissues. **(E)** Western blot of MPC2 protein in high-glucose (HG) treated cells with butyric acid (30/60 μM). **(F)** Immunofluorescence of MPC2 (red) and DAPI (blue) with Scale bars: 50 μm. The data represents at least three independent biological replicates (n ≥ 3), each containing three technical replicates. The data is presented as mean ± SD; Use independent sample t-test (or one-way ANOVA) for data that conforms to normal distribution; Non normally distributed data are analyzed using Mann Whitney U test (or Kruskal Wallis test) p < 0.05, **: p < 0.01, ***: p < 0.001.

Transmission electron microscopy (TEM) analysis of colonic tissues revealed profound mitochondrial abnormalities in diabetic mice, characterized by loss of elongated morphology, cristae disintegration, and blurred double-membrane boundaries (Figure 4A). Importantly, these pathological alterations—indicative of mitochondrial swelling and metabolic dysfunction—were significantly attenuated by LEO treatment at both 30 and 60 mg/kg doses, with high-dose administration restoring near-normal ultrastructure. Subsequent transcriptomic profiling comparing diabetic and LEO-treated (60 mg/kg) mice identified 11 differentially expressed mitochondrial genes (Figure 4B). Notably, LEO downregulated *ACADM*, *NDUFB8*, *SOD2*, *COX4I1*, and *SDHB* (involved in fatty acid oxidation and ROS detoxification), while concurrently upregulating *IDH3A*, GPX1, CPT1A, PDK4, and *MPC1/2* (critical for TCA cycle flux and pyruvate metabolism). Strikingly, *MPC2* exhibited the most pronounced upregulation. Given its established role as the gatekeeper of mitochondrial pyruvate import, this *MPC2* elevation implies enhanced substrate availability for oxidative phosphorylation, a process essential for energizing enteroendocrine functions.

Further supporting this mechanism, Western blot confirmed dose-dependent MPC2 protein upregulation in colons of LEO-treated mice (Figure 4C). To mechanistically link butyrate to this observation, we first demonstrated transcriptional activation in GLUTag cells, where butyrate (30 μM and 60 μM) significantly elevated MPC2 mRNA levels under HGHF stress (*p* < 0.01; Figure 4D). Subsequently, Western blot analysis further established dose-dependent induction of MPC2 protein expression, confirming translational regulation (Figure 4E). Most conclusively, immunofluorescence imaging revealed intensified MPC2 signal, verifying enhanced protein synthesis and subcellular targeting efficiency (Figure 4F). Collectively, butyrate-mediated upregulation of *MPC2* expression restores mitochondrial structure/function and enhances cellular energy metabolism. This facilitates oxidative phosphorylation, ultimately promoting *GLP-1* secretion in enteroendocrine cells, thereby delineating a key mechanism for LEO’s antidiabetic efficacy.

### 3.5 Butyrate-activated *MPC2* axis rescues mitochondrial energetics to potentiate *GLP-1* secretion in diabetic enteroendocrine cells

**FIGURE 5:**
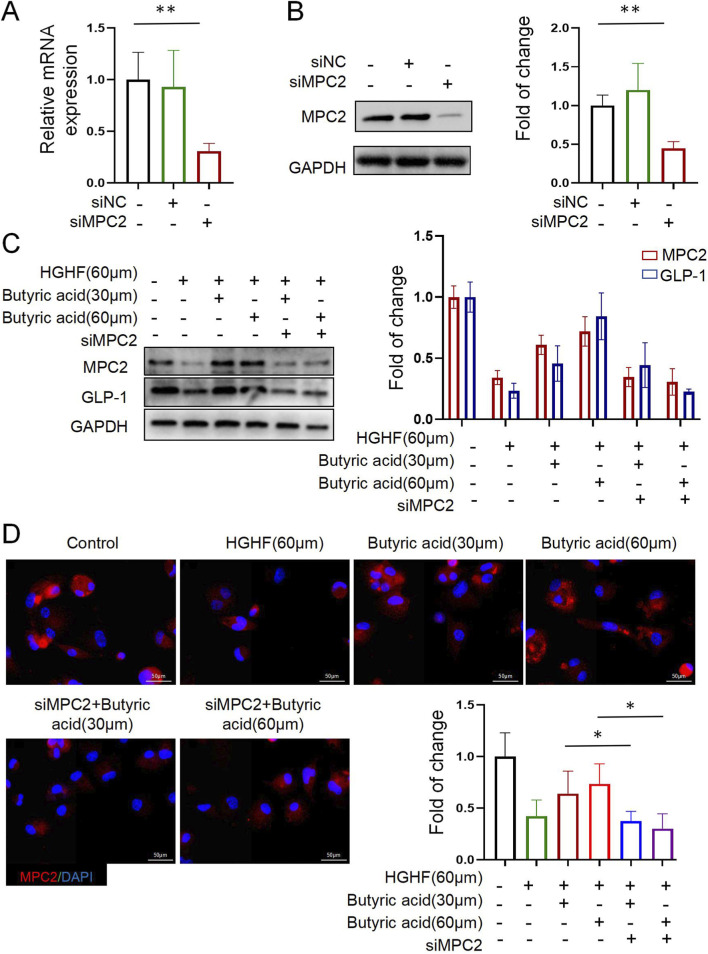
Genetic ablation of mitochondrial pyruvate carrier 2 (MPC2) abrogates butyrate-driven glucagon-like peptide-1 (GLP-1) secretion: Essential role in mitochondrial energetics. **(A)** MPC2 mRNA expression after siRNA transfection in GLUTag cells. **(B)** Western blot confirming MPC2 knockdown in cells. **(C)** Western blot confirming MPC2 and GLP-1 secretion fold change in cells treated with high-glucose (HG) and Butyric acid. **(D)** MPC2 and GLP-1 immunofluorescence in cells with scale bars: 50 μm. The data represents at least three independent biological replicates (n ≥ 3), each containing three technical replicates. The data is presented as mean ± SD; Use independent sample t-test (or one-way ANOVA) for data that conforms to normal distribution; Non normally distributed data are analyzed using Mann Whitney U test (or Kruskal Wallis test) p < 0.05, **: p < 0.01, ***: p < 0.001.

To establish whether butyrate stimulates GLP-1 secretion through MPC2, we performed loss-of-function studies using siRNA-mediated MPC2 knockdown in GLUTag cells. Transfection with siMPC2, but not scramble siRNA, significantly reduced both MPC2 mRNA and protein levels compared to controls (*p* < 0.01) (Figures 5A,B). Critically, under HG stress conditions, which suppressed both MPC2 and GLP-1 expression, subsequent treatment with butyrate (30 or 60 μM) dose-dependently rescued the expression of MPC2 and GLP-1 (*p* < 0.05 vs. HG alone). However, this rescue effect was completely abrogated by cotreatment with siMPC2 (p < 0.01 vs. butyrate alone) (Figure 5C). Immunofluorescence quantification further confirmed that butyrate-enhanced GLP-1 signal intensity was abolished upon MPC2 depletion (Figure 5D). Crucially, the suppression of GLP-1 by siMPC2 exhibited a clear concentration-dependence relative to the butyrate treatment: Notably, the inhibitory effect of siMPC2 on GLP-1 fluorescence was not more pronounced at the lower concentration of butyrate (30 μM) compared to the higher concentration (60 μM) (*p* < 0.001). This concentration-dependent reversal of butyrate’s effect by MPC2 knockdown definitively confirms MPC2 as the non-redundant conduit essential for butyrate’s action on GLP-1 secretion. Definitively, genetic silencing of MPC2 completely abrogated butyrate-induced potentiation of GLP-1 secretion, thereby establishing a causal “Gut Microbiota-Butyrate-MPC2-GLP-1” regulatory axis.

## 4 Discussion

Current research on the pathogenesis of T2D increasingly focuses on the “gut-islet axis,” where gut microbiota-derived SCFAs (Ng et al., 2022), particularly through promoting GLP-1 secretion, represent a significant therapeutic target (Zhuang et al., 2021; Yan et al., 2025). Gut microbiota has important role in the development of T2D and studies associates dysbiosis with IR and T2DM. SCFAs promotes intestinal barrier integrity, pancreatic β-cell proliferation and insulin biosynthesis, where enteric dysbiosis reduces SCFAs synthesis (Tan et al., 2014; Cunningham et al., 2021; Fusco et al., 2023). SCFAs and secondary bile acids improves insulin secretion, energy expenditure, and decrease lipogenesis (Zeng et al., 2024). Nevertheless, the precise molecular mechanisms through which microbial metabolites regulate enteroendocrine cell energy metabolism to drive GLP-1 release remain incompletely elucidated (Gribble and Reimann, 2019; Wang et al., 2023). Studies reports LEO’s potential in attenuating STZ-induced diabetic nephropathy via mitigating dyslipidemia and inflammation (Li et al., 2024). Critically, our research demonstrates that leonurine intervention enriches SCFA-producing genera while suppressing pro-inflammatory microbiota (Liao et al., 2021; Huang et al., 2025). Butyrate, a prominent SCFA, directly stimulates GLP-1 secretion from L cells, thereby improving hyperglycemia and insulin resistance in T2DM models (Sanna et al., 2019; Chen et al., 2021). This dual modulation of microbial ecology and host incretin physiology underscores leonurine’s unique therapeutic value. By synergistically targeting the “gut microbiota-SCFAs-GLP-1 axis”, leonurine represents a natural, orally administrable alternative to conventional therapies, circumventing their limitations.

This study is the first to systematically demonstrate that the botanical extract LEO ameliorates T2D metabolic disorder via a cascade mechanism: restructuring gut microbiota composition, restoring SCFA-producing bacterial populations, elevating colonic butyrate levels, activating MPC2 expression, and ultimately repairing mitochondrial energy metabolism in enteroendocrine cells to enhance GLP-1 secretion. Notably, in STZ-induced T2D mice, LEO intervention not only dose-dependently improved oral glucose tolerance (Figure 1B) but also significantly elevated GLP-1 levels in serum and colonic tissues. Critically, subsequent metagenomic analysis revealed that LEO (60 mg/kg) effectively reversed diabetes-associated dysbiosis, evidenced by a marked increase in α-diversity (Shannon index), elevated relative abundance of Firmicutes, and specific enrichment of butyrate-producing genera such as *Alistipes* (Figures 3B–E). These structural optimizations in the microbiota directly correlated with functional metabolic alterations, as LEO dose-dependently increased fecal concentrations of butyrate, acetate, and isobutyrate (Figures 3A–F). Importantly, butyrate was identified as the core effector molecule driving GLP-1 secretion, demonstrably activating GLP-1 release in GLUTag cells under HG conditions mimicking diabetic metabolic stress (Figures 3G–J).

Traditionally, mechanistic research on SCFA-mediated GLP-1 secretion, particularly for butyrate, has centered on cell membrane receptor pathways (e.g., GPR41/43/109a) or epigenetic regulation (HDAC inhibition) (Tolhurst et al., 2012; Kaji et al., 2014; Bolognini et al., 2021). However, the regulatory role of energy metabolism in enteroendocrine L-cells on GLP-1 synthesis and secretion has long been overlooked (Clara et al., 2016; Jorsal et al., 2018). Notably, GLP-1 release via vesicular exocytosis is highly ATP-dependent, and mitochondrial dysfunction—strongly linked to impaired gut hormone secretion in diabetes—manifests as ultrastructural abnormalities (e.g., cristae disintegration, matrix swelling) and reduced oxidative phosphorylation efficiency in T2D patients and animal models, decreasing ATP synthesis by 30%–40% (Sun et al., 2018; Liu et al., 2023; Yang et al., 2024). Although butyrate has been proposed to serve as an energy substrate via β-oxidation, the specific mechanisms by which microbial metabolites repair diabetes-associated mitochondrial damage and bridge “energy supply-hormone secretion” remained undefined (Tan et al., 2014; Cunningham et al., 2021; Fusco et al., 2023). In this context, the observed upregulation of MPC2 expression in our study holds pivotal significance: MPC2 acts as a critical node linking glycolysis to mitochondrial energy metabolism by transporting cytosolic pyruvate into mitochondria for oxidation. Importantly, LEO intervention, through elevating butyrate levels and synergistically upregulating MPC2, markedly enhanced mitochondrial pyruvate utilization capacity and overall ATP synthesis efficiency in enteroendocrine cells, thereby providing robust energetic support for sustained GLP-1 secretion. Crucially, this restoration of cellular energy metabolism plays a central role under the metabolically stressed environment induced by HG conditions. Nevertheless, the exact mechanism by which butyrate enhances MPC2 expression—remains to be elucidated.

Delving deeper into the mechanism, this study reveals MPC2-mediated mitochondrial functional repair as the central hub for butyrate-driven GLP-1 potentiation. Transmission electron microscopy (TEM) unequivocally demonstrated that LEO treatment effectively reversed pathological mitochondrial swelling and cristae disintegration in diabetic colonic tissue (Figure 4A). Subsequently, transcriptomic and proteomic validation confirmed that LEO significantly upregulated MPC2 expression in both colonic tissue and GLUTag cells (Figures 4B–F). Crucially, MPC2 functions as the key transporter regulating pyruvate entry into mitochondria; its activation directly enhances tricarboxylic acid (TCA) cycle flux and oxidative phosphorylation efficiency (Tavoulari et al., 2023; He et al., 2025). Most compellingly, MPC2 knockdown completely abrogated butyrate’s stimulatory effect on GLP-1 secretion (Figure 5). This pivotal finding definitively establishes a “Microbial Metabolite (Butyrate) – Mitochondrial Carrier (MPC2) – Gut Peptide Hormone (GLP-1)” causal regulatory axis, unveiling a novel mechanism by which the gut microbiota influences host glucose homeostasis through energy metabolism reprogramming. In metabolic diseases, MPC2 modulators are shown to offer a direct intervention for hepatic metabolic diseases (McCommis et al., 2017; Tavoulari et al., 2023). Prospects of MPC2 targeting are shown to be higher in obesity management, where MSDC-0602K, a next-generation thiazolidinedione in combinatory approaches is promising against diabetes (Colca et al., 2020; Kamm et al., 2021; Hodges et al., 2022). This combination of MSDC-0602K and the GLP-1 receptor agonist liraglutide produces synergistic benefits in this regard (Kamm et al., 2021), however, the downstream mechanisms connecting MPC2 activation to GLP-1 vesicular exocytosis are not fully addressed. Specifically, whether calcium signaling, ATP-sensitive potassium channels, or other pathways are involved remains undefined. Future studies should delineate the role of calcium signaling and KATP channels in MPC2-mediated exocytosis using specific pharmacological inhibitors and calcium imaging in GLUTag cells.

**FIGURE 6:**
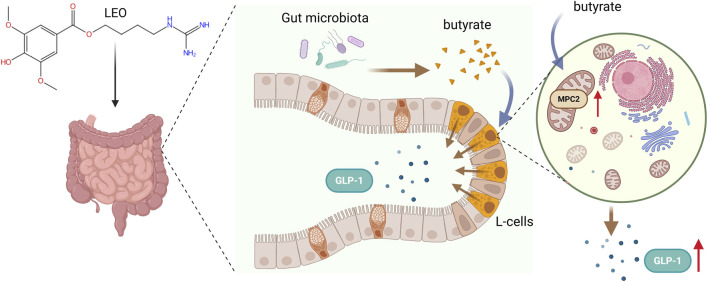
Mechanistic framework illustration through which leonurine ameliorates type 2 diabetes, showing gut microbiota remodeling, enhanced butyrate production, and mitochondrial pyruvate carrier 2 (MPC2) activation to restore glucagon-like peptide-1 (GLP-1) secretion.

This study systematically delineates the mechanistic framework through which the botanical extract LEO ameliorates glucose metabolic disorders in type 2 diabetes via multi-targeted cascading actions (Figure 6). Specifically, we demonstrate for the first time that LEO dose-dependently (30/60 mg/kg) restructures gut microbiota composition, selectively enriching butyrate-producing genera such as *Alistipes* and significantly elevating colonic butyrate concentrations to physiologically relevant levels. Interestingly, berberine, known for inducing GLP-1 and its remarkable antidiabetic properties, treatment has also shown to enrich *Alistipes* and enhance butyrate production (Wu et al., 2022; Duan et al., 2023; Araj-Khodaei et al., 2024). Further mechanistic investigation reveals that butyrate, functioning as the core effector molecule, activates mitochondrial pyruvate carrier MPC2 expression, effectively repairing ultrastructural damage in enteroendocrine cell mitochondria and enhancing tricarboxylic acid cycle flux to provide ATP energy essential for GLP-1 vesicular exocytosis. Collectively, these results solidify the scientific framework of LEO ameliorating diabetes via the gut microbiota-butyrate-MPC2 axis.

## 5 Conclusion

This work not only provides the molecular pharmacological basis for LEO’s anti-diabetic efficacy but more innovatively identifies MPC2 as a pivotal therapeutic target bridging gut microbial metabolism with enteroendocrine hormone secretion. This study lays the theoretical foundation for developing novel “mitochondrial energy reprogramming”-based interventions for diabetes. In conclusion, leveraging the proposed gut microbiota-SCFAs-GLP-1 axis offers a transformative approach for T2DM management. Nevertheless, certain limitations should be acknowledged: Firstly, the specific active constituents within LEO responsible for modulating the microbiota and MPC2 remain uncharacterized. Secondly, the downstream effectors linking MPC2 to GLP-1 secretion (e.g., calcium signaling or ATP-sensitive channels) require further elucidation y (He et al., 2025). Future investigations should utilize germ-free animal models and metagenomics sequencing approaches to validate the indispensability of the gut microbiota in this pathway, explore the synergistic therapeutic potential of MPC2-targeted agonists combined with LEO, and advance preclinical pharmacodynamics and safety evaluations. Therefore, further studies and clinical validations are warranted to translate these mechanistic insights into therapeutic applications.

## Data Availability

The datasets presented in this study can be found in online repositories. The names of the repository/repositories and accession number(s) can be found below: https://ngdc.cncb.ac.cn/omix/release/OMIX012113.
